# Short Link N promotes disc repair in a rabbit model of disc degeneration

**DOI:** 10.1186/s13075-018-1625-9

**Published:** 2018-08-29

**Authors:** Fackson Mwale, Koichi Masuda, Michael P. Grant, Laura M. Epure, Kenji Kato, Shingo Miyazaki, Kevin Cheng, Junichi Yamada, Won C. Bae, Carol Muehleman, Peter J. Roughley, John Antoniou

**Affiliations:** 1 Orthopaedics Research Laboratory, Lady Davis Institute for Medical Research – Jewish General Hospital, 3755 Chemin de la Côte-Sainte-Catherine, Montréal, QC H3T 1E2 Canada; 2 Department of Surgery, McGill University, 3655 Promenade Sir William Osler, Montréal, QC H3G 1Y6 Canada; 3 School of Medicine, University of California, 9500 Gilman Dr., La Jolla, CA 92093 USA; 4 Department of Biochemistry, Rush University Medical Center, 600 S Paulina St, Chicago, IL 60612 USA; 5 Shriners Hospital for Children, McGill University, 1003 Decarie Blvd, Montréal, QC H4A 0A9 Canada

**Keywords:** Intervertebral disc degeneration, Regeneration, Tissue engineering, Collagen, Proteoglycans, Bioactive peptides, Short link N

## Abstract

**Background:**

The degeneration of the intervertebral disc (IVD) is characterized by proteolytic degradation of the extracellular matrix, and its repair requires the production of an extracellular matrix with a high proteoglycan-to-collagen ratio characteristic of a nucleus pulposus (NP)-like phenotype in vivo. At the moment, there is no medical treatment to reverse or even retard disc degeneration. The purpose of the present study was to determine if a low dose of short link N (sLN), a recently discovered fragment of the link N peptide, could behave in a manner similar to that of link N in restoring the proteoglycan content and proteoglycan-to-collagen ratio of the disc in a rabbit model of IVD degeneration, as an indication of its potential therapeutic benefit in reversing disc degeneration.

**Methods:**

Adolescent New Zealand white rabbits received an annular puncture with an 18-gauge needle into two noncontiguous discs to induce disc degeneration. Two weeks later, either saline (10 μL) or sLN (25 μg in 10 μL saline) was injected into the center of the NP. The sLN concentration was empirically chosen at a lower molar concentration equivalent to half that of link N (100 μg in 10 μL). The effect on radiographic, biochemical and histologic changes were evaluated.

**Results:**

Following needle puncture, disc height decreased by about 25–30% within 2 weeks and maintained this loss for the duration of the 12-week study; a single 25-μg sLN injection at 2 weeks partially restored this loss in disc height. sLN injection led to an increase in glycosaminoglycans (GAG) content 12 weeks post-injection in both the NP and annulus fibrosus (AF). There was a trend towards maintaining control disc collagen-content with sLN supplementation and the GAG-to-collagen ratio in the NP was increased when compared to the saline group.

**Conclusions:**

When administered to the degenerative disc in vivo, sLN injection leads to an increase in proteoglycan content and a trend towards maintaining control disc collagen content in both the NP and AF. This is similar to link N when it is administered to the degenerative disc. Thus, pharmacologically, sLN supplementation could be a novel therapeutic approach for treating disc degeneration.

## Background

Adjacent vertebrae are linked together by the fibrocartilaginous intervertebral discs (IVDs), which endow the spine with its flexibility and resistance to compression [1]. IVDs are composed of highly organized concentric fiber-reinforced lamellae layers rich in collagen termed the annulus fibrosus (AF), and a central gel-like and pressurized nucleus pulposus (NP) consisting mainly of randomly oriented collagen fibrils and high concentrations of the proteoglycan aggrecan [2, 3]. The ability to withstand compression is provided by the high negative-charge density of the glycosaminoglycan (GAG) chains attached to aggrecan monomers [4, 5]. In the human, the IVDs are separated from the vertebral bone by the cartilage endplates at the superior and inferior boundaries, which are a major pathway of nutrient supply into the IVD [6, 7]. These substructures provide the disc’s mechanical function to support and distribute large and multi-directional spine loads and deformations.

One of the factors that lead to degeneration of the IVD is a reduction in nutrient supply. Calcification of the endplate has been considered to play a significant role as a barrier to nutrient transport into the IVD. Furthermore, the functional ability of the IVD is impaired due to decreases in GAG content, which occurs relatively early during human aging [8, 9]. During IVD degeneration, dramatic changes occur in the NP as it becomes more fibrous and loses the delineation between the NP and the inner AF [3, 10]. At later stages of degeneration, the collagen fibrils of the NP become damaged, leading to the formation of clefts that eventually also affect the AF. This degeneration of IVD structure and function is a progressive condition and presents a major socioeconomic burden worldwide because of its strong association with back pain. The most recent Global Burden of Disease study identified back pain as the single most common cause of disability worldwide [11]. Other contributors to disc degeneration are biochemical [12–16], biomechanical [17–20] and genetic [21–24]. Historically, disc degeneration has been assumed to be the result of aging and “wear and tear” from mechanical insults and injuries. However, the Twin Spine Study has recently suggested that IVD degeneration may be due, in large part, to genetically determined “developmental” changes in disc structure [25].

Spinal fusion surgery is a commonly performed surgical procedure for the treatment of degenerative disc disease (DDD) with severe pain [26]. While this may provide symptomatic relief, studies have demonstrated that spinal fusion surgery accelerates disc degeneration in adjacent vertebral segments [27]. Ideally, biological repair of the degenerating IVD would be preferable to fusion, though at present there are no disease-modifying medications yet available.

Link N is a naturally occurring 16 amino acid peptide representing the N terminal region of link protein, a glycoprotein that stabilizes proteoglycan aggregates in IVD and cartilage by binding to both hyaluronic acid and aggrecan [28]. The peptide is generated by matrix metalloproteinases (MMPs) during tissue turnover in vivo [29] and has been shown to enhance proteoglycan synthesis by IVD cells and intact human discs, and also to increase disc height in a rabbit disc puncture degeneration model [30]. It is postulated that these biochemical changes are due to link N interacting with the BMP type II receptor on disc cells and activating intracellular Smad1/5 signaling [31]. Hence, link N may provide therapeutic benefit to degenerating discs. Two major advantages of link N over recombinant growth factors are that it is more economically viable and does not stimulate bone formation [31]. Recently, we found that the first 1–8 residues of link N (short link N, sLN) are responsible for the biological activity [32].

The purpose of the present study was to determine whether a low dose of sLN could promote disc repair by assessing proteoglycan content and the proteoglycan-to-collagen ratio in a rabbit model of IVD degeneration.

## Methods

### Peptide synthesis

Short link N (sLN) DHLSDNYT (link N 1–8) with a mass of 964 Da was synthesized by CanPeptide (Pointe Claire, QC, Canada).

### Surgical procedure for the rabbit IVD degeneration model

**Fig. 1:**
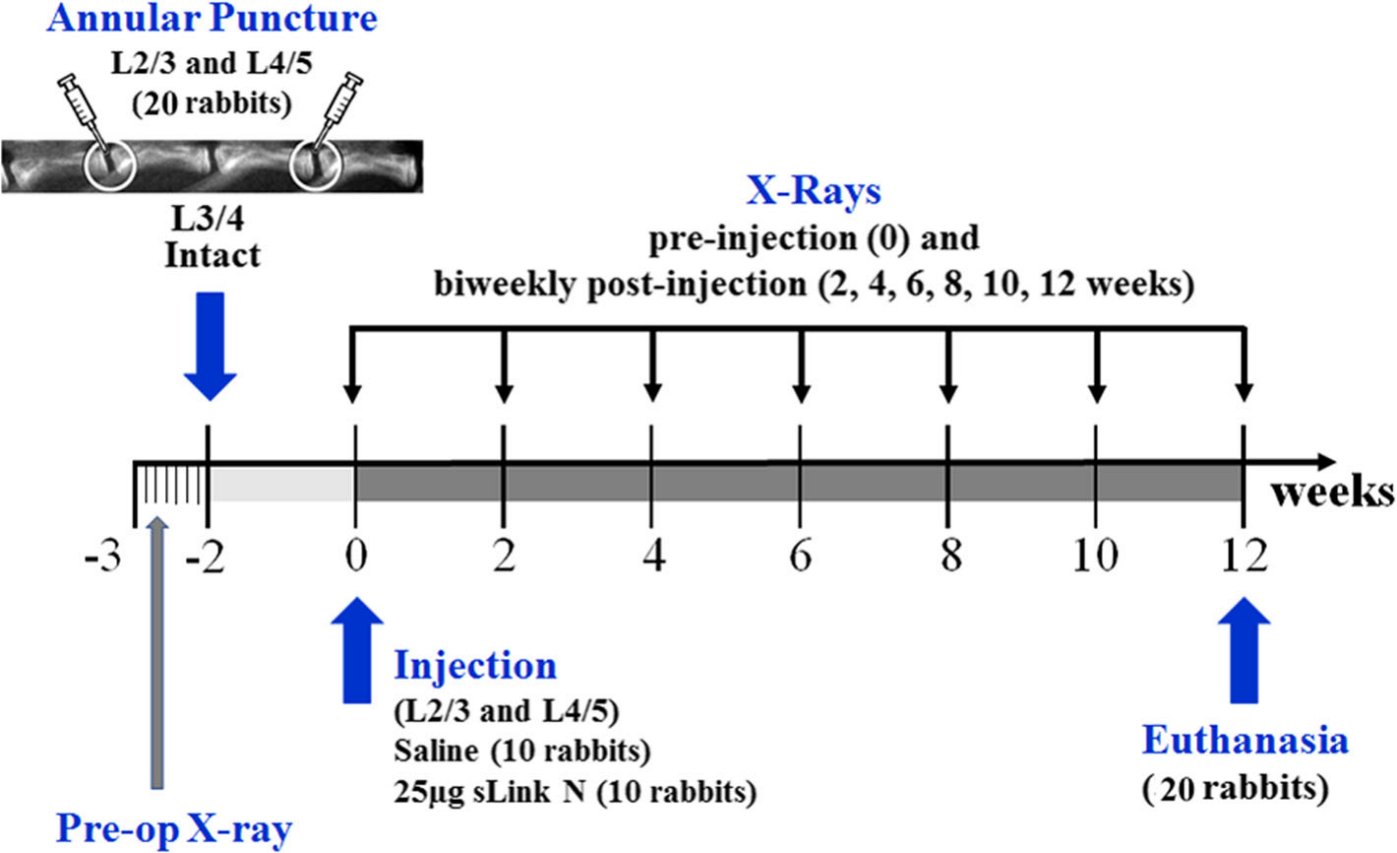
Schematic of disc puncture model timeline and short link N (sLink N) treatment. Pre-op, preoperative

Twenty New Zealand White rabbits (specific pathogen-free (SPF), 4–6 month-old skeletally mature rabbits, Western Oregon Rabbit Co. OR, USA) weighing approximately 3.5 kg were used in the present study (Fig. 1) with the approval of the Institutional Animal Care and Use Committee, La Jolla, CA, USA. Five days before surgery, a pre-operative x-ray was taken as a baseline control under anesthesia by the intramuscular administration of ketamine hydrochloride (25 mg/kg) and acepromazine maleate (1 mg/kg, 10 mg/mL). IVD degeneration was induced in all rabbits as previously described [30]. Briefly, the rabbits were placed in a lateral prone position and the anterior surfaces of three consecutive lumbar IVDs (L2/3, L3/4 and L4/5) were exposed through a posterolateral retroperitoneal approach by blunt dissection of the psoas muscle. IVD degeneration was induced at the L2/3 and L4/5 levels, by AF puncture in the ventral aspect into the NP using an 18G needle with a stopper device that allows the needle to penetrate to a maximum depth of 5 mm. The IVD at the level L3/4 was left intact and used as a non-punctured control. The surgical wound was repaired in layers and the skin was closed using staples. Intraoperative and postoperative x-rays were taken to confirm the level of puncture. After recovery from anesthesia, the rabbits were returned to their cages and mobilized ad libitum. The x-rays confirmed the presence of degenerated discs, 2 weeks post-surgery.

### sLN injection

Two weeks after the initial surgery, both punctured discs (L2/3 and L4/5) of each rabbit were injected intradiscally through their anterolateral surfaces into the NP area with either saline (10 μL/disc) or 25 μg sLN dissolved in 10 μL saline as indicated in Table 1, using a MS*GFN25 microsyringe equipped with a XX*MS16 needle (Ito Corporation, Fuji, Japan) (Fig. 1). In our previous study with full length link N, we used 100 μg per disc [30]. The non-punctured disc (level L3/4) of each rabbit remained untreated and was used as an internal control for IVD repair assessment. After intradiscal injection, the rabbits were returned to their cages and closely monitored for behavior, appetite and change in body weight.

**Table 1:** Number of rabbits and discs used in the study to evaluate the effect of sLink N on the repair of the degenerative IVDs

	Number of rabbits	Number of punctured discs (L2/3, L4/5)	Number of intact discs^a^ (L3/4)	Total rabbits per analysis
Biochemistry				
Saline	7	14	7	14
sLN	7	14	7	
Histology				
Saline	3	6	3	6
sLN	3	6	3	
Total rabbits^b^				20

*sLN* short link N

^a^Intact discs (L3/4) were used as internal control

^b^All rabbits (intervertebral discs) were used for biweekly X-ray measurement of disc height evaluation

### Radiographic analysis of IVD height

Radiographs were taken of all rabbit spines in a flexed position, using a digital radiography system (resolution 71 μm; NAOMI, Nagano, Japan) at biweekly intervals up to 12 weeks after disc puncture (Fig. 1). A consistent level of anesthesia (ketamine hydrochloride 25 mg/kg and acepromazine maleate 1 mg/kg) was maintained during radiography of each animal and at each time point to obtain a similar degree of muscle relaxation, which may affect the disc height. To decrease the error from axial rotation of the spine and from beam divergence, radiographs were repeated until a complete lateral image was obtained on each animal in the lateral decubitus position with the beam centered at 4 cm from the rabbit iliac crest. An orthopedic researcher who was blinded to the study group, surgical procedure and time point independently interpreted all x-ray images. All radiograph images were analyzed using a custom program for MATLAB software (Natick, MA, USA).

IVD height, expressed as the disc height index (DHI = IVD height/adjacent vertebral body height), was based on the previously developed method [33]. The mean DHI was calculated by averaging the height measurements obtained from the anterior, middle and posterior portions of the IVD and dividing that by the average of the adjacent vertebral body heights. Changes in the DHI of injected discs were expressed as percentage DHI (%DHI) and normalized to the measured non-punctured control DHI (normalized %DHI = [punctured DHI/non-punctured DHI] × 100). The preoperative x-ray was always used as a baseline measurement: %DHI = (postoperative DHI/preoperative DHI) × 100.

### Euthanasia and sample preparation

At 12 weeks after treatment, rabbits in each group were anesthetized with ketamine hydrochloride (25 mg/kg) and acepromazine maleate (1 mg/kg) and euthanatized with an excess dose of pentobarbital (Euthanasia B solution: Henry Schein Inc., Melville, NY, USA). Rabbit discs were excised from spines and either prepared for histological evaluation or dissected into NP and AF tissues for biochemical analysis (Table 1).

### Histological assessment of the IVD

Rabbit IVDs containing part of the vertebrae were fixed in 10% neutralized formalin, decalcified with Cal-Ex™ II Fixative/Decalcifier and embedded in paraffin. Sagittal 4-μm sections of each IVD were dewaxed in Safeclear™ Tissue Clearing Agent (ThermoFisher Scientific, USA) and stepwise rehydrated in ethanol: 100%, 95%, 70%, 50%, distilled water. For proteoglycan content, the sections were stained with 1% Alcian blue 8GX (pH 1.0) for 30 min and then counterstained in 0.1% Nuclear Fast red for 5 min. Sections were dehydrated and mounted in Permount (ThermoFisher Scientific, USA). Images were captured on a Leica DM LB2 light microscope (Leica Microsystems GmbH, Wetzlar, Germany).

### Biochemical analysis

Treated discs from each experimental group were removed from each lumbar spine for biochemical analysis (Table 1). Discs from six additional control rabbits were also analyzed. After disc excision, the NP was separated from the AF. All specimens were weighed (wet weight) and digested with proteinase K at 56 °C for 48 h. The proteinase K tissue digests were quantified for sulfated glycosaminoglycans (GAG) and total collagen content.

### GAG analysis

Sulfated glycosaminoglycans (GAGs) were quantified in tissue extracts by a modified dimethyl methylene blue (DMMB) dye-binding assay [34, 35]. Samples were diluted to fall within the middle of the linear range of the standard curve. Extraction buffer of an equal volume to the tissue extracts was added to the standard curve to compensate for possible interference.

### Hydroxyproline content

The proteinase K tissue digests were used for analysis of hydroxyproline as a measure of total collagen content. For analysis, digests were first hydrolyzed in 6 M HCl to release free hydroxyproline, which was then quantitated using dimethylaminobenzaldehyde [36]. Total collagen content was estimated assuming that hydroxyproline content is equivalent to 10% of the weight of each collagen α chain [37].

### Statistical analysis

Statistical analysis was performed using the GraphPad Prism (version 6.03; GraphPad Software, Inc., La Jolla, CA, USA) program. Differences between the sLN-injected and saline-injected groups were assessed with one or two-way repeated analysis of variance (ANOVA) and post hoc Tukey’s multiple comparisons test.

## Results

**Fig. 2:**
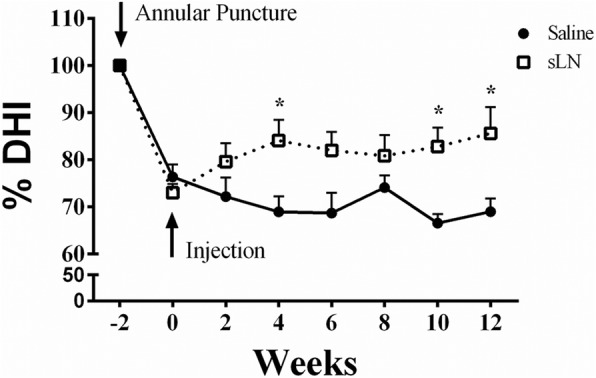
Effect of short link N (sLN) on disc height index (DHI). Two weeks following annular puncture, rabbits were injected with saline or sLN. DHI was measured every 2 weeks by radiography and converted to a percentage of disc height index (%DHI) using the adjacent non-punctured disc as a control. Symbols represent means ±SEMs. Two-way analysis of variance and post hoc Tukey’s multiple comparisons test was used to compare all groups. Comparison between saline and sLN, *p < 0.05, at each time point; *n* = 10

Following needle puncture, the normalized %DHI decreased by about 25–30% over the next 2 weeks compared with the baseline DHI values obtained before puncture. This loss stabilized and there was no recovery over the 12-week period (Fig. 2). A single 25-μg sLN injection appeared to begin reversing the loss of disc height during the first 2 weeks, and by week 4 the mean normalized %DHI of injected discs in the sLN group was statistically higher than that in the saline group. This difference was maintained during the next 8 weeks with the mean normalized %DHI of injected discs in the sLN group being significantly higher than that in the saline group at 12 weeks after the sLN 25-μg injection.

**Fig. 3:**
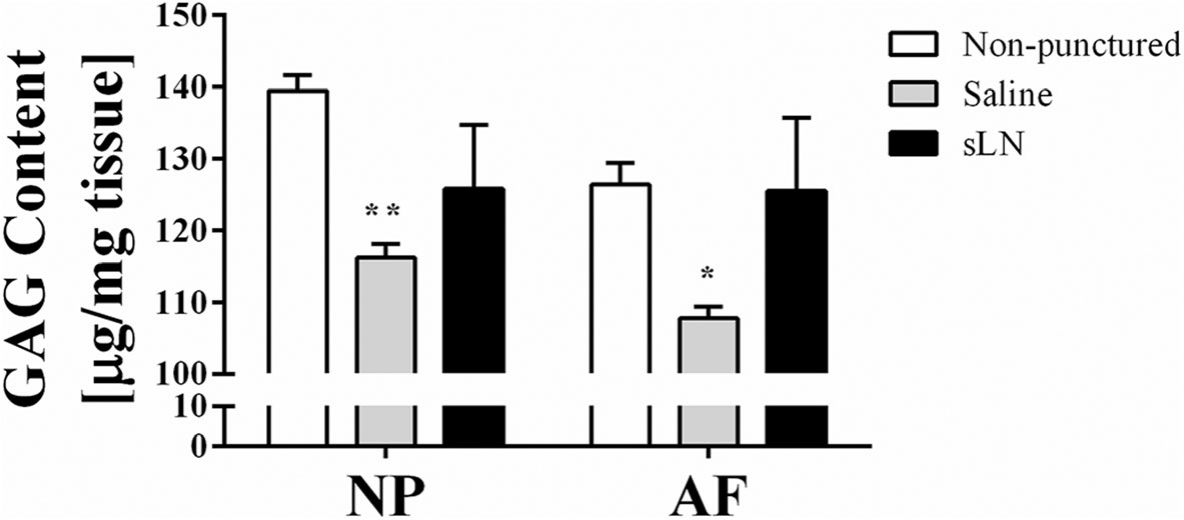
Proteoglycan content in intervertebral discs (IVDs) treated with short link N (sLN). After 12 weeks rabbits were sacrificed and IVDs were extracted and dissected into nucleus pulposus (NP) and annulus fibrosus (AF) tissues. Glycosaminoglycan (GAG) content was taken as a measurement of proteoglycan in the NP and AF. Means ± SEMs. Analysis of variance and post hoc Tukey’s multiple comparisons test was used to compare all groups. Comparison between saline and sLN to non-punctured discs, *p < 0.05, **p < 0.01; *n* = 3–7

GAG concentration was measured in the discs because of the major role proteoglycans play in the functional ability of IVDs to swell and resist compressive forces [38]. There was a significant decrease in GAG content in the NP and AF of discs from the saline group when compared to the non-punctured discs after 12 weeks, indicative of proteoglycan (aggrecan) loss (Fig. 3). sLN stimulated an increase in GAG content in both the NP and AF.

**Fig. 4:**
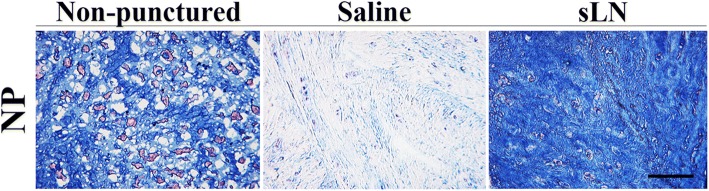
Histological assessment for proteoglycan content in the intervertebral discs (IVDs). Histological staining of rabbit IVDs using Alcian Blue to demonstrate changes in proteoglycan content in the nucleus pulposus (NP). Nuclear Fast red was used as a counterstain to visualize cells. Images were captured using a × 20 objective. Scale bar represents 100 μm. sLN, short link N

Alcian blue was used to stain sections of the disc for histological analysis in order to study proteoglycan content and distribution in the NP (Fig. 4). The blue color intensity decreased in the NP of punctured discs (saline-treated) when compared to the non-punctured control. In contrast, sLN supplementation led to an increase in the intensity of the blue color, which was distributed uniformly throughout the NP. The Alcian blue staining in the sLN-treated disc was comparable to non-punctured discs confirming increased proteoglycan content and uniform distribution throughout the NP. However, reduced Alcian blue staining does not indicate absence of proteoglycan in the tissue but rather a reduced content.

**Fig. 5:**
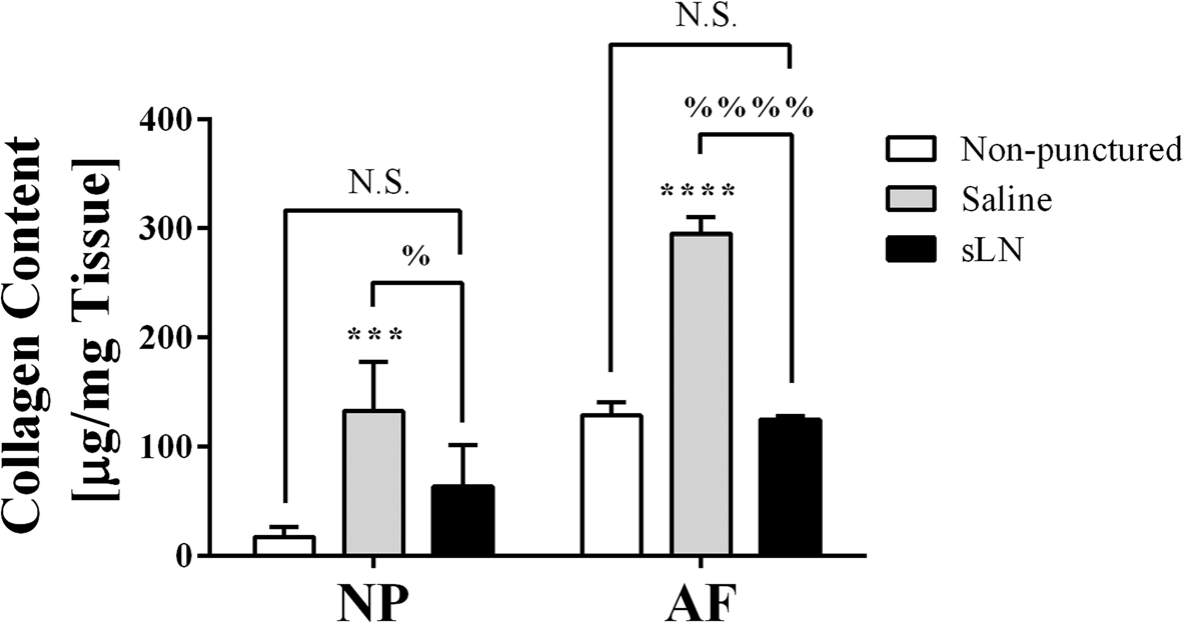
Collagen content in the intervertebral discs (IVDs) treated with short link N (sLN). The nucleus pulposus (NP) and annulus fibrosus (AF) of the dissected IVDs were measured for total collagen content using the hydroxyproline assay. Means ± SDs. Analysis of variance and post hoc Tukey’s multiple comparisons test was used to compare all groups. Comparison of saline and sLN to non-punctured discs, **p < 0.01, ****p < 0.0001; saline to sLN, %, *p* < 0.05, %%%%, *p* < 0.001, N.S., not significant; *n* = 3–7. GAG, glycosaminoglycans

Hydroxyproline as an indicator of disc collagen content was measured because of the intimate role that collagen plays in the mechanical strength of IVDs and its resistance to swelling. The discs responded to AF puncture by significantly increasing collagen content in both the NP and AF when compared to the non-punctured discs (*p* < 0.01 in the NP; *p* < 0.001 in the AF) (Fig. 5). Treatment with sLN maintained collagen content in both NP and AF tissues to levels comparable to non-punctured discs.

**Fig. 6:**
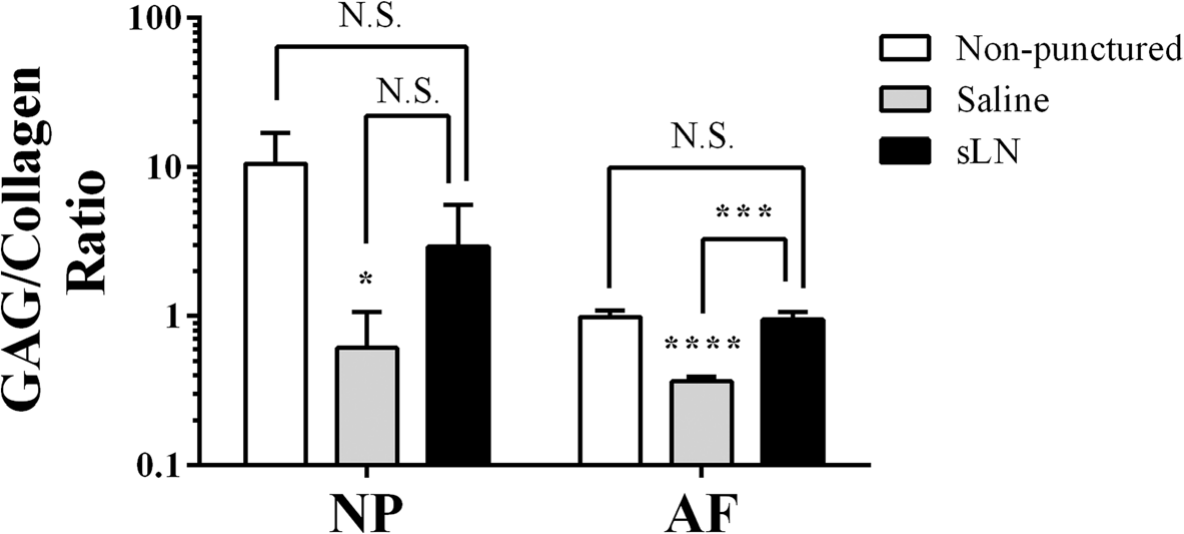
Proteoglycan/collagen ratio in the intervertebral discs (IVDs). The proteoglycan/collagen ratio was calculated from the glycosaminoglycan and hydroxyproline data presented in Figs. 3 and 5, respectively. Means ± SDs. Analysis of variance and post hoc Tukey’s multiple comparisons test was used to compare all groups. Comparison of saline and sLN to non-punctured discs, *p < 0.05, ***p < 0.001, ****p < 0.0001, N.S., not significant; *n* = 3–7. NP, nucleus pulposus; AF, annulus fibrosus; sLN, short link N

We have previously shown that an extracellular matrix with a high proteoglycan-to-collagen ratio can be used as a means to identify a NP-like phenotype in vivo [9]. In non-punctured discs with no degeneration, the GAG-to-collagen ratio in the NP was on average 10.5:1, while that of the AF was 1.0:1 (Fig. 6). Following needle puncture, the GAG-to-collagen ratios in the NP and AF decreased significantly to an average of 0.9:1 and 0.4:1, respectively. For the sLN treated groups, the GAG-to-collagen ratio in the NP increased when compared to the saline group to an average of 3.0:1 while that of the AF was increased to 0.9:1.

## Discussion

Previous studies have shown that link N can stimulate the proteoglycan content and increase disc height when administered to the degenerative rabbit disc in vivo [30] . The present data indicate that sLN is as good as link N in stimulating the proteoglycan content in this model at half the molar mass. The changes in proteoglycan and disc height shown here with sLN are also similar to those observed with bone morphogenetic protein 7 (BMP7) [39]. The increase in disc height observed was not linear. There seems to be a plateau effect in about 6–8 weeks. It is unclear whether this is of any significance in the repair process or just reflects the natural variation in disc height measurements.

Injection of several growth factors, such as BMP-7, transforming growth factor-β (TGFβ), fibroblast growth factor (bFGF), growth/differentiation factor 5 (GDF-5) into the disc has been widely studied as a means to stimulate extracellular matrix production and cell proliferation [39–42]. Intradiscal administration of recombinant human BMP-7 in a rabbit model of disc degeneration led to an increased disc height and enhanced proteoglycan content of the NP [39, 42], showing its potential to promote repair in disc degeneration. However, others have found extensive extradiscal bone formation in a spontaneous canine IVD degeneration model treated with BMP-7 [43]. Thus sLN appears to be as effective as other growth factors in stimulating repair of the IVD in vivo, but in common with link N does not induce bone formation [44]. A second major advantage of sLN over a growth factor such as BMP-7 for therapeutic use is that is simple and cost-effective to manufacture.

In the present work, we wanted to see if sLN would perform the same as link N in terms of in disc repair. We used a molar concentration (25 μg) half of that used previously with long link N to determine whether lower doses can improve disc repair. Without sLN treatment there was a significant decrease in GAG content in the NP and AF with loss of disc height, indicative of proteoglycan degradation and loss. After sLN treatment there was both an increase in disc height and an increase in GAG concentration, indicative of new proteoglycan synthesis in both the NP and AF.

In contrast, a significant increase in collagen content was observed throughout the disc during degeneration in the absence of sLN. This can be explained in part by the decrease in disc volume due to compression. However, as disc height decreases by 25%, it is likely that new collagen synthesis must also be occurring. With a 25-μg sLN injection, the collagen content decreases relative to degeneration alone, which could be due partly to re-swelling and partly to diminished synthesis. Since collagen is initially deposited during the 2-week degeneration period, some collagen may remain in the NP even following sLN treatment due to its slow removal during tissue remodeling.

The present histological evaluation using Alcian blue staining indicates that the non-punctured NP matrix and that treated with sLN is Alcian blue positive. It was reported that in a relatively non-degenerated human Pfirrmann grade-2 disc, the NP matrix is uniformly positive for Alcian blue [45]. In a mildly degenerated Pfirrmann grade-3 disc and in a more degenerated Pfirrmann grade-4 disc, the NP matrix is more fibrous and the Alcian blue reactivity is significantly reduced, suggesting that degeneration is associated with an alteration in proteoglycan content. The increase in Alcian blue staining with sLN is consistent with increased proteoglycan content and disc height, suggesting that sLN mitigates further degeneration and stimulates repair. Interestingly, although NP cellularity is initially affected following induced degeneration in our puncture model, cellularity was preserved following sLN treatment unlike the saline controls as observed from our histological sections. In the absence of sLN, cellularity appeared to diminish as the degeneration cascade was allowed to progress; in the presence of sLN, cellularity was maintained, due to the inhibition of cell death.

## Conclusions

In conclusion, sLN injection can stimulate proteoglycan production in vivo in both the NP and AF similar to link N when it is administered to the degenerative disc. Thus, pharmacologically sLN supplementation could be a potential therapeutic approach for treating disc degeneration.

## Abbreviations

AF: Annulus fibrosus
BMP: Bone morphogenic protein
DDD: Degenerative disc disease
DHI: Disc height index
GAG: Glycosaminoglycans
IVD: Intervertebral disc
MMP: Matrix metalloproteinase
NP: Nucleus pulposus
sLN: Short link N
